# Activating Old Materials with New Architecture: Boosting Performance of Perovskite Solar Cells with H_2_O‐Assisted Hierarchical Electron Transporting Layers

**DOI:** 10.1002/advs.201801170

**Published:** 2018-12-21

**Authors:** Fengyou Wang, Meifang Yang, Yuhong Zhang, Lili Yang, Lin Fan, Shiquan Lv, Xiaoyan Liu, Donglai Han, Jinghai Yang

**Affiliations:** ^1^ Key Laboratory of Functional Materials Physics and Chemistry of the Ministry of Education Jilin Normal University Changchun 130103 China; ^2^ College of Physics Jilin Normal University Siping 136000 China; ^3^ National Demonstration Center for Experimental Physics Education Jilin Normal University Siping 136000 China; ^4^ School of Materials Science and Engineering Changchun University of Science and Technology Chuangchun 130022 China

**Keywords:** electron transporting layers, hierarchical mesoporous architecture, high power conversion efficiency, perovskite solar cells

## Abstract

The breakthrough of organometal halide perovskite solar cells (PSCs) based on mesostructured composites is regarded as a viable member of next generation photovoltaics. In high efficiency PSCs, it is crucial to finely optimize the charge dynamics and optical properties matching between the perovskites and electron transporting materials to relax the trade‐off between the optical and electrical requirements. Here, a simple antipolar route with H_2_O as the additive is proposed to prepare hierarchical electron transporting layers to boost the efficiency of dopant‐free PSCs. The photovoltaic performance of the PSCs is enhanced owing to increased light‐scattering, improved Ostwald ripening, and photo‐generated electron extraction. Optimization of the H_2_O addition enables a valid power conversion efficiency of 19.9% (reverse scan: 20.02%) to be achieved. The device can retain more than 90% of its initial performance after storage in air more than 30 days. These results are inspiring in that they present that a mesoporous transporting layer could be easily re‐constructed to hierarchical architecture by the antipolar method to further improve the performance of PSCs.

## 1 Introduction

Inorganic–organic hybrid perovskite solar cells (PSCs) have attracted considerable attention owing to their unique characteristics such as the broad range in which they absorb sunlight, low exciton binding energy, long electron–hole diffusion length, tunable direct band gaps, and high extinction coefficient.^1^, ^2^, ^3^, ^4^ These cells, which have an n‐i‐p architecture, can be fabricated by using different hole transporting layers (HTLs) and electron transporting layers (ETLs) on the rear and front sides of the perovskite layer, respectively, to selectively extract the photo‐generated carriers to an external circuit. The highest power conversion efficiency (PCE) of (FAPbI_3_)_0.95_(MAPbBr_3_)_0.05_ solar cells is 23.2%.^5^ The preparation process and the dose of each cation used as dopant need to be sophisticatedly controlled with the view of achieving ideal crystallization and good chemical stability. Therefore, it is difficult to maintain a high level of reproducibility. In contrast, the reproducibility of robustly fabricated dopant‐free MAPbI_3_‐based solar cells is easier to control. However, owing to their modest ability to extract photo‐generated carriers extraction and their wider bandgap than FA‐doped perovskite materials, which always result in a lower short circuit current density, the PCE of MAPbI_3_‐based solar cells is still not optimal and needs to be further improved.^6^ The ETL is important because it not only provides the path for electron extraction, but also has a significant impact on the light‐scattering characteristics and chemical nucleation of the perovskite layers. Therefore, it is necessary to consider the optical and electrical, as well as the chemical and physical stability of ETLs simultaneously to realize a high‐performance device. Previously, materials such as TiO_2_, PCBM, SnO_2_, or ZnO were widely adopted into PSCs to enhance electron extraction and transport. Among all these alternative ETLs, mesoporous TiO_2_ (m‐TiO_2_) is the most commonly used n‐type semiconductor and acts as scaffold layer for a large portion of PSCs, owing to its favorable band‐edge positions, superior chemical stability, and low cost. However, a major drawback of planar m‐TiO_2_ ETLs, which renders them unsuitable for application as PSCs, is that their interfacial charge extraction is insufficiently rapid. The charge accumulation at the TiO_2_/perovskite interface induced by ion migration in the perovskite layer is known to cause hysteresis, thereby causing the performance of the device to deteriorate.^7^, ^8^ To solve this problem, we previously modified planar TiO_2_ ETLs with a thin PC_61_BM buffer layer, and found that the elastic nature of the PC_61_BM could facilitate the formation of high‐quality perovskite films and improve the TiO_2_/perovskite interfacial properties.^9^ Considering the modest efficiency of these solar cells and the high cost of the PC_61_BM, it is still essential as well as vital to explore a more comprehensive way to boost the charge extraction and performance of these solar cells. Enlarging the TiO_2_/perovskite interfacial area may effectively release the accumulated electrons at the hetero‐interface because this would increase the probability of electron extraction by the ETL.^10^, ^11^ This suggests that suitably shaping the architecture of ETLs may be an alternative approach to optimize the charge dynamics at the TiO_2_/perovskite hetero‐interface to improve the performance of the PSCs.

Compared with traditional planar m‐TiO_2_ ETLs, the hierarchical structure of TiO_2_ consists of particles of different sizes that could provide a larger interfacial area for charge extraction and more flexible architecture for light‐scattering. In addition, as the substrate of perovskite layer, morphological properties of hierarchical structure TiO_2_ (size, distribution, and structure, etc.) can be modulated efficiently; hence, conceptually TiO_2_ is a more likely candidate for controlling the nucleation of the perovskite. Thus, in principle, hierarchical TiO_2_ could be an effective solution for improving the performance of the device.^12^, ^13^, ^14^ Previously, hierarchically structured TiO_2_, which is synthesized by a two‐step hydrothermal procedure, was used as the ETL in liquid state dye‐sensitized solar cells to enhance the performance of the device.^15^ Nevertheless, for PSCs, owing to the lack of appropriate architectural modulation, introducing a substrate with hierarchical architecture usually reduces the crystallinity of the perovskite films, which increases the density of defects and TiO_2_/perovskite interfacial recombination. Therefore, the use of hierarchically structured TiO_2_ to enhance the performance of PSCs beyond that of T‐m‐TiO_2_‐based PSCs has not yet been reported.

Accordingly, in this work, we propose a tri‐functional hierarchical m‐TiO_2_ (H‐m‐TiO_2_) ETLs, which is first fabricated using H_2_O as an additive to shape the architecture and balance the light‐trapping, crystallization, and charge extraction properties. The MAPbI_3_ solar cells with the n‐i‐p structure and H‐m‐TiO_2_ ETLs show a high efficiency of 20.02% (reverse scan) and are almost free of hysteresis. The enhanced performance is attributable to the unique H‐m‐TiO_2_ ETLs, which improves the light‐scattering behavior and crystallization of the perovskite layer, and accelerates charge extraction. This work not only demonstrates a simple route for preparing hierarchical carrier transporting layers, but also paves the way for the possible application of hierarchical ETLs in extensive optoelectronic devices.

## 2 Results and Discussion

**Figure 1:**
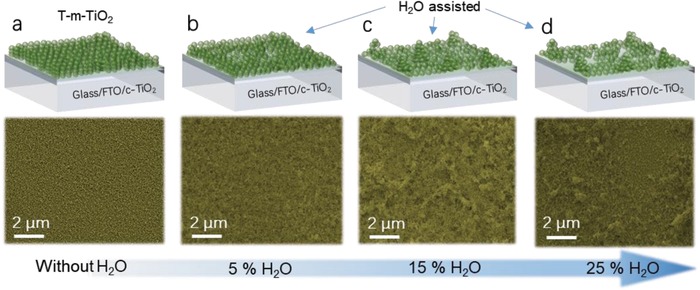
Schematic diagrams and SEM images of m‐TiO_2_ films with different H_2_O addition: a) 0%, b) 5%, c) 15%, and d) 25%. The white dash circles show the areas without m‐TiO_2_.

First, we examine the morphological changes upon H_2_O addition (**Figure** **1**a–d). Top‐view scanning electron microscopy (SEM) images of m‐TiO_2_ films with 0, 5, 15, and 25% v/v of added H_2_O are shown in Figure 1a–d, respectively. The traditional m‐TiO_2_ (T‐m‐TiO_2_) film without H_2_O addition shows a flat porous structure, which coincides with the state‐of‐the‐art results that were previously reported.^16^, ^17^, ^18^ As the addition of H_2_O increase to 15%, the roughness of the surfaces of the m‐TiO_2_ films increases and some microscale pits on the original m‐TiO_2_ are created (H‐m‐TiO_2_). This can be ascribed to the large differences in polarity between the H_2_O and the solvent of the TiO_2_ colloid (ethanol), which provides sufficient surface tension to allow assembling of the TiO_2_ particles. Large amounts of TiO_2_ nanoparticles are accumulated to form the clusters, whereas particles in other areas become thinly spread as a consequence. Increasing the addition of H_2_O even further to 25% causes too many TiO_2_ particles to wash out during the spin‐coating process, resulting in the exposure of large areas of the compact TiO_2_ films.

**Figure 2:**
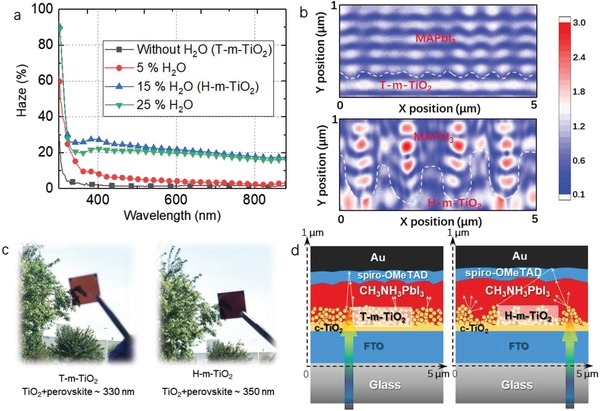
The optical properties of the H‐m‐TiO_2_ ETLs. a) Haze factor of m‐TiO_2_ layers with various H_2_O addition from 0% to 25%. b) The simulated intensity distribution of electric fields of 600 nm wavelength incident light within the device on (top) T‐m‐TiO_2_ ETLs and (bottom) H‐m‐TiO_2_ ETLs. c) Photographs of MAPbI_3_ films on T‐m‐TiO_2_ ETLs and H‐m‐TiO_2_ ETLs. d) Schematics of incident light paths within MAPbI_3_ solar cells based on T‐m‐TiO_2_ or H‐m‐TiO_2_ substrates. The concept of light‐trapping in solar cell is illustrated by the arrows representing incoming and scattered sunlight.

Empirically, different morphological characteristics are likely to affect the optical properties of the ETLs. As the window layer of n‐i‐p type PSCs, surface texturization is the preferable configuration to ensure effective light‐trapping over a wide wavelength range.^19^, ^20^, ^21^ We examined the optical properties of the ETLs, including their vertical transmittance (VT) and total transmittance (TT) by UV–Vis–NIR spectrophotometry (Figure S1, Supporting Information). Increasing of H_2_O addition from 0 to 15% causes the VT to reduce, whereas the TT almost remains unchanged (Figure S2a,b, Supporting Information), indicating that the surface light‐scattering properties of the surface are promoted. However, when the addition of H_2_O is promoted to 25%, the VT only increases slightly because the TiO_2_ films are partially washed out (as shown in Figure 1d). The haze factor is one of the major roles to evaluate the light‐scattering ability of the substrate. We calculated the haze factor from the variation between VT and TT (**Figure** **2**a) as follows:^22^

(1) $$\mathrm{Haze}=\frac{\left(T\;T-V\;T\right)}{T\;T}\times 100$$

Without the addition of H_2_O, T‐m‐TiO_2_ films could not produce an adequate light‐scattering effect in the wavelength region of 400–800 nm owing to the small feature size of mesoporous structure. This means that much of the light is transmitted directly by the substrate without changing its orientation. After constructing the hierarchical structure, large TiO_2_ clusters were able to effectively scatter the incident light, thereby resulting in an increased haze factor (Figure 1c). Optical simulations of the PSCs prepared on T‐m‐TiO_2_ and H‐m‐TiO_2_ ETLs were carried out to ascertain their light harvesting capability. The shape parameters that were used for the optical simulation are exhibited in Figure S3 in the Supporting Information. Figure 2b shows the 2D optical intensity distribution of electric fields in the perovskite layer. Detailed information of the simulation is included in Note S1 in the Supporting Information. Incident light with a wavelength of 600 nm incident from the fluorine‐doped tin oxide (FTO) side was used for the simulation, and the color scale bars illustrating the absorption intensity were confined to the same range. It shows that for perovskite films on T‐m‐TiO_2_ ETLs, a hierarchical electric field which originated from the forward‐ and reverse‐propagating light waves exists in perovskite absorber layer, indicating moderate light‐scattering. In comparison, strong light‐scattering, owing to the larger surface roughness of the H‐m‐TiO_2_ ETLs, is found in perovskite films, which results in more intense absorption. Figure 2c shows images of the perovskite thin films grown on T‐m‐TiO_2_ ETLs and H‐m‐TiO_2_ ETLs, respectively. Obviously, the H‐m‐TiO_2_ ETLs sample exhibits a darker color, indicating stronger absorption than the T‐m‐TiO_2_ ETLs sample. The light paths are schematically depicted in Figure 2d to visually illustrate benefits of this light‐trapping effect for PSCs. Once the incident light approaches the H‐m‐TiO_2_ ETLs, the stronger light‐scattering effect (higher haze factor) between the H‐m‐TiO_2_ and MAPbI_3_ films prolongs the light path. Consequently, compared with T‐m‐TiO_2_ ETLs, MAPbI_3_ films based on H‐m‐TiO_2_ are expected to achieve stronger light absorption (Figure S4, Supporting Information). In addition, for full cells with spiro‐OMeTAD and Au layers on top, the absorption of the cell could be further enhanced due to the subsequent larger reflection angle at the interfaces of MAPbI_3_/spiro‐OMeTAD/Au.

**Figure 3:**
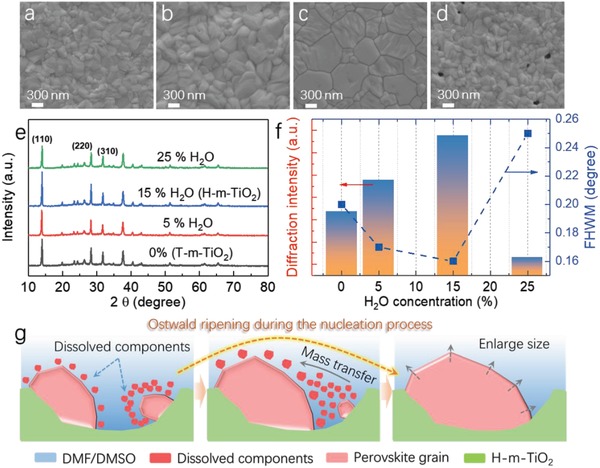
SEM images of the MAPbI_3_ films on different TiO_2_ substrate: a) T‐m‐TiO_2_, b) 5% H_2_O addition, c) 15% H_2_O addition (H‐m‐TiO_2_), and d) 25% H_2_O addition. e) XRD patterns, f) FWHM and diffraction intensity of the MAPbI_3_ films on different TiO_2_ substrates. g) Schematic diagram of the Ostwald ripening procedures during the nucleation process.

Except for the optical behavior, the crystallization of perovskite films based on various substrates was investigated. Cuboid MAPbI_3_ crystals between 210 and 500 nm are formed on the pristine T‐m‐TiO_2_ ETLs (**Figure** **3**a). For the substrates evolving from T‐m‐TiO_2_ to H‐m‐TiO_2_, as the addition of H_2_O increases from 0% to 15%, this also causes the grain size of MAPbI_3_ films to increase (Figure 2b,c). However, some pinholes and small grains are formed in the MAPbI_3_ films, thereby further escalating the H_2_O addition of the m‐TiO_2_ substrates. The X‐ray diffraction (XRD) patterns of the MAPbI_3_ films also verify the crystallization features (Figure 3e,f). All compositions exhibit a typical perovskite peak at ≈14°, which corresponds to the (110) orientation of the photoactive black phase of MAPbI_3_. Compared with the other samples, the films grown the on the H‐m‐TiO_2_ achieve the highest diffraction intensity and lowest full width at half maximum (FWHM), which also indicates improved crystallization. All these results imply that the crystallization characteristics of perovskite films seem to be greatly affected by the architecture of the substrates.

Here, we introduce the Ostwald ripening model to unravel the coarsening of the perovskite particles. During the nucleation process, precursors with different particle sizes are initially formed on the H‐m‐TiO_2_ surface. The relationship between the chemical potential and particles radius can be depicted as follows:^23^

(2) $$\mu =\mu_{0}+\frac{2\beta V}{r}$$

where μ is the chemical potential of the surface, β is the surface energy, μ_0_ is the chemical potential for a flat surface, *r* is the radius of a particle, and *V* is mole volume of a particle. Hence, a smaller particle is energetically less stable than a larger particle owing to higher chemical potentials. This means small grains are easily dissolved than large grains if there is a sufficient amount of solvent present. The dissolved components between large grain and small grain will lead to a concentration gradient, causing the mass transportation from small grain to large grain according to Fick's first law.^24^ Therefore, as the period of annealing is elongated, the small grains vanish and the size of the large grains increases because of the mass transportation of the dissolved component. In this case, compared with the flat T‐m‐TiO_2_ layer, the H‐m‐TiO_2_ layer, which consists of small nanoscale mesoporous and large microscale holes, would require less solvent (DMF and DMSO) after the spin coating process. During the heating procedure, the H‐m‐TiO_2_ layer would inevitably delay the extraction of solvent, and prolong the ripening of the MAPbI_3_ precursor to form larger columnar crystals. Hence, the large perovskite grains finally coarsen by absorbing surrounding small grains via mass transportation (Figure 3g). In contrast, for the T‐m‐TiO_2_ layer or the non‐optimized layers (with 5% or 25% H_2_O added), due to its flatter architecture, the solvent molecules are extracted more easily from the precursor films, which leave less time for Ostwald ripening process.

**Figure 4:**
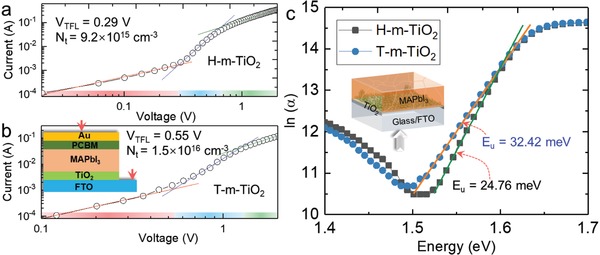
The effect of H‐m‐TiO_2_ and T‐m‐TiO_2_ on the defects density of the perovskite films. Current–voltage characteristics of the device with different types of m‐TiO_2_ configuration: a) H‐m‐TiO_2_ and b) T‐m‐TiO_2_, respectively. c) ln(α) as a function of energy for MAPbI_3_ films based on H‐m‐TiO_2_ and T‐m‐TiO_2_ ETLs, respectively.

Considering that crystallization always determines the defects in perovskite films, we also investigated the trap state density and band tail state density in T‐m‐TiO_2_‐based and H‐m‐TiO_2_‐based perovskite films. We studied the dark current–voltage characteristics and light‐absorption characteristics for electron‐only devices. **Figure** **4**a,b illustrates the dark current–voltage characteristics of the solar cells, indicating linear ohmic response at low bias, a trap‐filling regime, and a trap‐free space charge limit current (SCLC) regime. The trap state density was determined by the trap‐filled voltage as follows:^25^

(3) $$N_{t}=\frac{2\varepsilon_{0}\varepsilon_{r}V_{\mathrm{TFL}}}{qL^{2}}$$

where ε_r_ is the relative dielectric constant, ε_0_ is the vacuum permittivity, *V*_TFL_ is the onset voltage of the trap‐filled limit region, *L* is the thickness of the film, and *q* is the elemental charge. We found that the trap densities remarkably decrease from 1.5 × 10^16^ cm^−3^ to 9.2 × 10^15^ cm^−3^ for T‐m‐TiO_2_ ETLs and H‐m‐TiO_2_ ETLs, respectively. We also examined the Urbach energy (*E*_u_) of the films, which probe the sharp onset of absorption at the direct bandgap and deliver significant details regarding disorders in shallow energy level (Figure 3b). The *E*_u_ for perovskite films are calculated as follows:^26^

(4) $$\alpha =\alpha_{0}\;e^{\frac{E}{E_{u}}}$$

where α_0_ is constant, α is the absorption coefficient, and *E* is the photonic energy. We observed lower *E*_u_ values for the H‐m‐TiO_2_ sample, which indicates a smaller degree of electronic disorder at the band‐edge. These results imply that inducing the H‐m‐TiO_2_ ETLs could effectively suppress the defects in the MAPbI_3_ films due to the enhanced crystallization.

**Figure 5:**
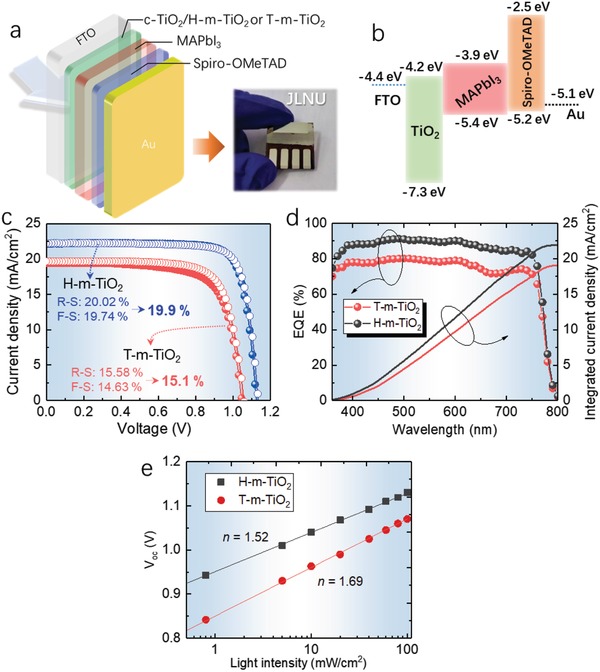
a) Architecture and b) energy bandgap diagrams of the MAPbI_3_ solar cells. Inset (a): the graph of the practical devices (aperture area: 0.1 cm^2^). c) Illuminated *J–V* curves (F‐S and R‐S) and PCE of the solar cells. The open circles indicate reverse scan data, and the filled circles represent forward scan data. d) EQE and integrated current density of the MAPbI_3_ solar cells. e) Dependence of the *V*_oc_ of H‐m‐TiO_2_ and T‐m‐TiO_2_‐based solar cells on the light intensity. Lines show linear fits to the experimental data.

The dependence of the performance of the PSCs on the T‐m‐TiO_2_ and H‐m‐TiO_2_ ETLs were studied in detail to further examine the application potential and develop a comprehensive understanding of the merits of H‐m‐TiO_2_ in PSCs. The fabrication process of the PSCs is demonstrated in Figure S5 in the Supporting Information. **Figure** **5**a,b shows the structure and energy bandgap of the PSCs. The photocurrent density–voltage (*J–V*) characteristics, including the forward scan (F‐S) and reverse scan (R‐S) of the studied PSCs, are shown in Figure 5c. For the solar cells with T‐m‐TiO_2_ ETLs, the PCE of R‐S and F‐S are 16.0% (R‐S: *V*_oc_ = 1.07 V, *FF* = 76.3%, *J*_sc_ = 19.6 mA cm^−2^) and 15.04% (F‐S: *V*_oc_ = 1.05 V, *FF* = 73.5%, *J*_sc_ = 19.5 mA cm^−2^), respectively. The difference in the PCE between F‐S and R‐S is 0.96%. As expected, the champion cells based on H‐m‐TiO_2_ ETLs exhibit weaker hysteresis behavior. The PCE of R‐S and F‐S is 20.02% (R‐S: *V*_oc_ = 1.13 V, *FF*= 80.2%, *J*_sc_ = 22.1 mA cm^−2^) and 19.74% (F‐S: *V*_oc_ = 1.13 V, *FF* = 79.8%, *J*_sc_ = 21.9 mA cm^−2^), respectively. The PCE difference between F‐S and R‐S is 0.28%, which gives a valid PCE of 19.9%. To our best knowledge, this is a very high valid efficiency for dopant‐free MAPbI_3_‐based solar cells. Figure 4d shows the external quantum efficiency (EQE) and integrated current density of the solar cells. Apparently, the cells based on H‐m‐TiO_2_ have superior spectra response in the wavelength range from 350–800 nm. The integrated *J*_sc_ of the solar cells is 21.9 mA cm^−2^ (H‐m‐TiO_2_) and 19.6 mA cm^−2^ (T‐m‐TiO_2_), respectively. Furthermore, we also investigated the reproducibility of this technique. The photovoltaic parameters of 40 devices (20 cells based on H‐m‐TiO_2_, 20 cells based on T‐m‐TiO_2_) prepared in one batch are summarized in Figure S6 in the Supporting Information. The average PCE of 19.4% for these 20 H‐m‐TiO_2_ devices can be achieved, which is also higher than the devices based on T‐m‐TiO_2_ ETLs.

These results indicate that the performance enhancements of the solar cells can be ascribed to the synergistic effect among different properties. First, the improved light‐scattering characteristics via the hierarchical architecture could elevate the light absorption of the MAPbI_3_ films by extending the effective light path. Therefore, the spectra response of the solar cells based on H‐m‐TiO_2_ would be better in a large wavelength region (Figure 5d). Second, as shown in Figures 3a–f and 4, introducing the H‐m‐TiO_2_ ETLs could enhance the crystallization of the MAPbI_3_ films and reduce the grain boundaries and trap‐states density. To obtain deeper insights into the impact of different ETLs on the trap‐states and charge‐dynamics of the device, the influence of the logarithm of light intensity *P* on the *V*_oc_ characteristics was investigated (Figure 5e). This logarithmic relationship is consistent with the fundamental relationship *V*_oc_ = *nk*_B_ *T/q* ln(*P*), where *n* is a constant referred as the ideality factor, *k*_B_ is Boltzmann's constant, *T* is the temperature, and *q* is the elementary charge.^27^, ^28^ According to the literature, the *P* dependence of the *V*_oc_ can provide insights into the role of trap‐assisted recombination versus bimolecular recombination at open circuit.^29^, ^30^ The recombination processes always strongly determine the properties of solar cells at open circuit condition because there is no current extraction and all the photo‐generated carriers recombine. It is generally recognized that the ideality factor should be equal to 1 if Langevin recombination dominates, whereas other involvements of the interfacial trap‐assisted Shockley–Read–Hall recombination would result in *n* being larger than 1. In this case, the value of *n* obtained for T‐m‐TiO_2_ and H‐m‐TiO_2_ is 1.69 and 1.52, respectively. This implies that the trap‐assisted Shockley–Read–Hall recombination is present in both devices. In addition, the smaller *n* of the H‐m‐TiO_2_‐based solar cells also suggests that improved crystallization reduces recombination and consequently leads to the improvement of the photovoltaic performance.

**Figure 6:**
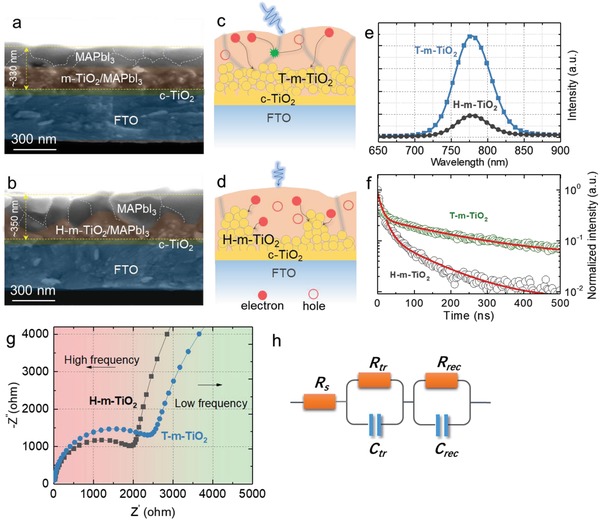
SEM images (cross‐section) of the MAPbI_3_ solar cells based on a) T‐m‐TiO_2_ and b) H‐m‐TiO_2_ ETLs, respectively. Schematic diagram of the charge extraction of c) T‐m‐TiO_2_ ETLs and d) H‐m‐TiO_2_ ETLs, respectively. Carrier transfer characterization: e) Steady state PL spectra and f) TRPL decay transient measured for FTO/H‐m‐TiO_2_ (or T‐m‐TiO_2_)/MAPbI_3_. g) Nyquist plots of the PSCs based on T‐m‐TiO_2_ and H‐m‐TiO_2_ ETLs. h)The equivalent circuit model of fitted EIS.

Moreover, as previously reported, a good electronic contact with faster extraction could reduce charge accumulation at the MAPbI_3_/contact layer interface induced by ion migration, and could promote carrier transportation.^9^, ^31^, ^32^, ^33^ Here, we suppose that the vertical architecture (with a large surface area) of the H‐m‐TiO_2_ may form an additional radial collection path for photo‐generated charges (**Figure** **6**a,b), which may accelerate the extraction of photo‐generated charges (Figure 6c,d). To confirm the enhanced charge extraction by H‐m‐TiO_2_ ETLs, we characterized the devices by steady photoluminescence (PL) and time‐resolved photoluminescence (TRPL) (Figure 6e,f). More intensive PL quenching and a shorter lifetime would indicate faster charge transfer at the H‐m‐TiO_2_/MAPbI_3_ interface. This accelerating photo‐generated electron extraction could effectively reduce the accumulation of charge at the hetero‐interface, separate the holes and electrons, eliminate the recombination, and suppress the hysteresis behavior of the device. To gain more insight on the charge transport and recombination at the interface, electrical impedance spectroscopy (EIS) measurements were also performed in the as‐fabricated PSCs (Figure 6g). Nyquist plot of EIS spectra were measured under dark and open circuit conditions, in the frequency range from 0.1 MHz to 10 Hz. As shown in Figure 6e, the fitted equivalent circuit model is composed of a series resistance (*R*_s_), charge transfer resistance (*R*_tr_) at the ETLs/MAPbI_3_ and the MAPbI_3_/spiro‐OMeTAD interfaces, and recombination resistance (*R*_rec_) forming a parallel circuit with capacitors (*C*_tr_ and *C*_rec_). The fitted parameters of *R*_s_, *R*_tr_, and *R*_rec_ are exhibited in Table S1 in the Supporting Information. The *R*_s_ is obtained from the *x*‐axis intercept of the high‐frequency curve. *R*_tr_ is ascribed to the high‐frequency arc and *R*_rec_ is assigned to low‐frequency arc. The values of *R*_s_ show little variation for these two devices, indicating that the hierarchical TiO_2_ ETLs have no significant effect on the series resistance. The H‐m‐TiO_2_‐based device exhibits a lower value of *R*_tr_, probably because the hierarchical architecture could extract the charge effectively. These results could be further verified by applying 0.8 V bias voltage to the device (Figure S7, Supporting Information). Thus, *n* carriers could be more easily extracted to the external circuit because charges are not trapped in the trapping centers in the perovskite films. Therefore, the H‐m‐TiO_2_ layer could not only improve the light‐scattering behavior and crystallization of the MAPbI_3_'s crystallization, but also enhance the extraction of photo‐generated charges.

**Figure 7:**
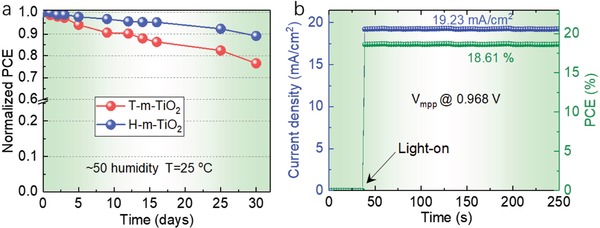
Stability of the PSCs with different ETLs. a) Device performances as a function of storage time in an ambient environment (≈50% humidity, *T* = 25°C). b) Stabilized maximum power point voltage, current density, and PCE.

In addition, as an emerging technique for PSCs, it is necessary to evaluate the device stability to assess the application potential. PSCs are known to be sensitive to humid conditions. Furthermore, the heat produced by light illumination cannot be quickly and effectively spread out generally in the encapsulated PSCs, which could be an alternative reason for the instability of inorganic–organic perovskite materials. We, therefore, examined the stability of the PSCs (30 days, under ambient conditions, 50% humidity) based on both T‐m‐TiO_2_ ETLs and H‐m‐TiO_2_ ESLs (**Figure** **7**a). The performance (90% of the pristine PCE) of the devices based on H‐m‐TiO_2_ ETLs was superior. We attribute this to the enhanced crystallization of the MAPbI_3_ films, which resist the moisture penetration during the aging process. Figure 7b shows the stabilized outputs of PSCs at the voltage of maximum power point (*V*_mpp_) under continuous irradiation. Both current and PCE of the champion cell change very little after soaking under one‐sun for 250 s, which demonstrates the good irradiation stability of the devices.

## 3 Conclusion

In summary, we successfully demonstrated solution‐processed H‐m‐TiO_2_ ETLs for high‐performance MAPbI_3_ solar cells. The resultant H‐m‐TiO_2_ ETLs, which were prepared by a H_2_O‐assisted method, are highly textured, and confer efficient light‐trapping properties upon the PSCs. Benefiting from its unique architecture, the crystallization of MAPbI_3_ is improved owing to the enhanced Ostwald ripening process. Photo‐generated charge extraction from MAPbI_3_ is also promoted due to the additional radial collection induced by H‐m‐TiO_2_ ETLs. Therefore, the tri‐functional H‐m‐TiO_2_ ETLs can provide respectable photoelectrical enhancement to yield a high valid PCE of 19.9% (R‐S: 20.02%, F‐S: 19.74%) in the derived devices. In addition, a compact and stable protecting layer for device is spontaneously formed to enhance the ambient stability of the derived PSCs. About 90% of its initial PCE can be retained after 30‐day exposure in ambient conditions with ≈50% relative humidity. These H‐m‐TiO_2_ ETLs with hierarchical architecture not only provide a simple way to improve the performance and stability of PSCs, but also shows the great advantages in further development of efficient optoelectronic devices.

## 4 Experimental Section

*Fabrication of H‐m‐TiO_2_ ETLs*: FTO glass substrates in the dimension of 2 × 2 cm^2^ were patterned by etching with zinc powder and 2 m hydrochloric acid. The substrates were in sequence washed by ultrasonication with soap (5% Hellmanex in water), absolute alcohol, acetone, and deionized water for 20 min, and then cleaned by UV‐ozone for 20 min. A 30 nm‐thick layer of dense compact TiO_2_ was coated onto the FTO‐glass by spin‐coating at 2000 rpm for 30 s and then annealed at 135 °C for 10 min. The TiO_2_ paste (Dyesol 18NR‐T) was diluted with ethanol with weight ratio of 1:7. Then, different volume ratios (0%–25%) of deionized water were dripped in the mixed solution. The solution was stirred in room temperature for 1 h. The as‐prepared solution was spin‐coated onto the TiO_2_ compact film at 5000 rpm for 30 s to fabricate H‐m‐TiO_2_ layers. These H‐m‐TiO_2_ layers were first dried at 135 °C for 10 min and then sintered at 500 °C for 30 min in air atmosphere.

More details about the materials and PSCs fabrication procedure are demonstrated in Notes S2–S4, Supporting Information.

## Conflict of Interest

The authors declare no conflict of interest.

## Supporting information

Supplementary
