# Supplementary material for: Activating Old Materials with New Architecture: Boosting Performance of Perovskite Solar Cells with H2O‐Assisted Hierarchical Electron Transporting Layers
Source: Adv Sci (Weinh). 2018 Dec 21;6(4):1801170. doi: 10.1002/advs.201801170 (PMC6382312; doi:10.1002/advs.201801170)
Supplement: Supplementary file 1 — Supplementary [file ADVS-6-1801170-s001.pdf]

Copyright WILEY-VCH Verlag GmbH & Co. KGaA, 69469 Weinheim, Germany, 2018.

ADVANCED SCIENCE Open Access

Supporting Information

for *Adv. Sci.,* DOI: 10.1002/advs.201801170

Activating Old Materials with New Architecture: Boosting Performance of Perovskite Solar Cells with H_2O-Assisted Hierarchical Electron Transporting Layers

Fengyou Wang, Meifang Yang, Yuhong Zhang, Lili Yang, Lin Fan, Shiquan Lv, Xiaoyan Liu, Donglai Han, and Jinghai Yang**

Copyright WILEY-VCH Verlag GmbH & Co. KGaA, 69469 Weinheim, Germany, 2016.

Supporting Information

Activating old materials with new architecture: boosting performance of perovskite solar cells with H_2O-assisted hierarchical electron transporting layers

Fengyou Wang, Meifang Yang, Yuhong Zhang, Lili Yang*, Lin Fan, Shiquan Lv, Xiaoyan Liu, Donglai Han, Jinghai Yang*

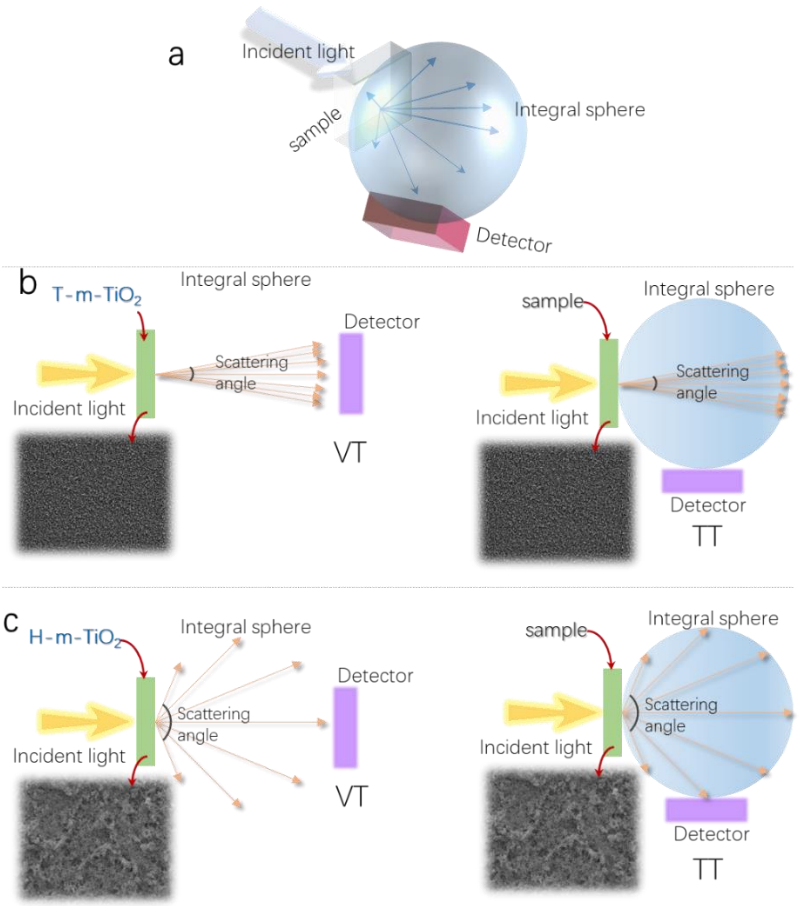

Figure S1. (a) schematic presentation of the optical measurement system. VT and TT test models for the (a) T-m-TiO_2 and (b) H-m-TiO_2 substrates.

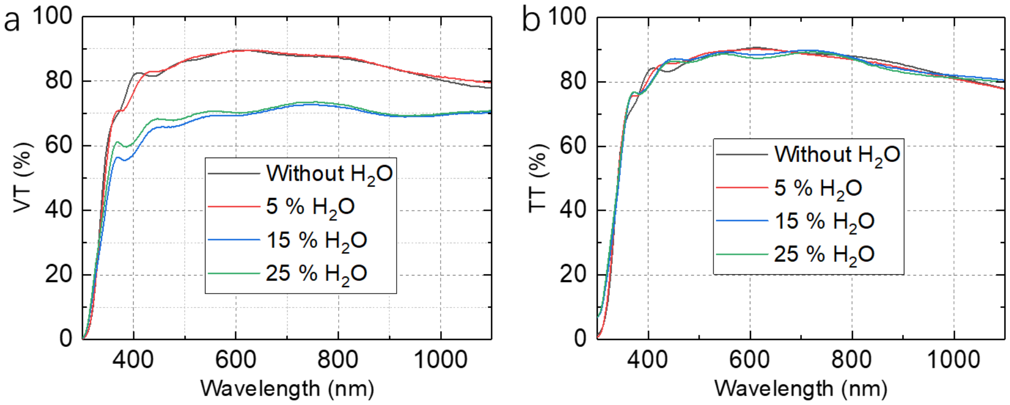

Figure S2. The VT and TT spectra of the ETLs with different H_2O-addition.

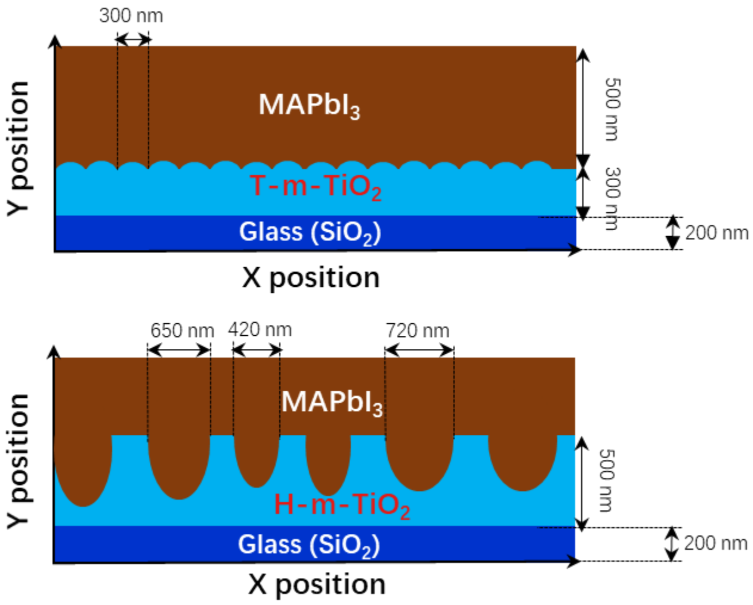

Figure S3. Schematic diagram of the simulated model. The input shape parameters are also illustrated. The glass (SiO_2) is 200 nm (not shown in the Fig. 2b of the manuscript). The T-m-TiO_2 is constructed by TiO_2 film (thickness~300 nm) and TiO_2 sphere (diameter~300 nm); The H-m-TiO_2 is constructed by TiO_2 films (thickness~500 nm) and $MAPbI_3$ ellipsoid (random size~400 nm-700 nm). The $MAPbI_3$ ellipsoids are used to etch the TiO_2 to form a random roughness.

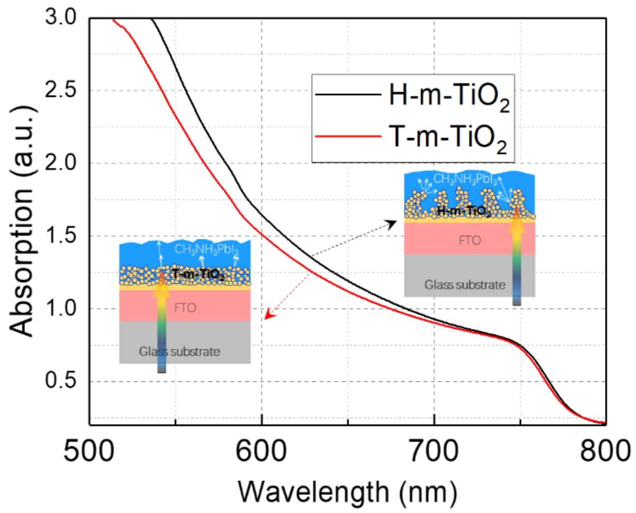

Figure S4. Optical absorption of the $MAPbI_3$ films grown on different substrate.

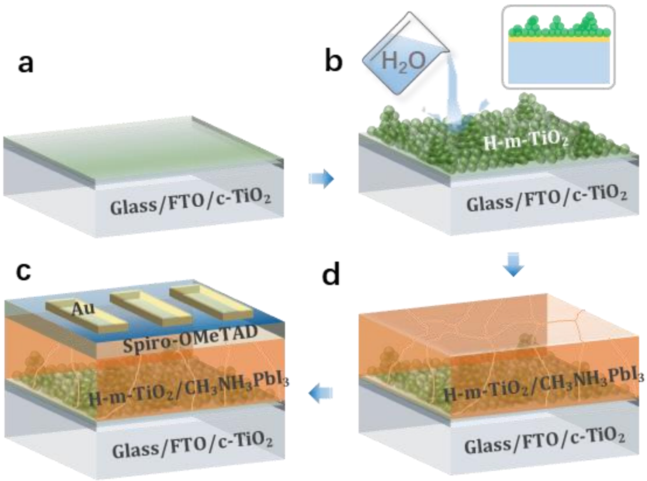

Figure S5. schematic diagram of the preparation procedure of the perovskite solar cells

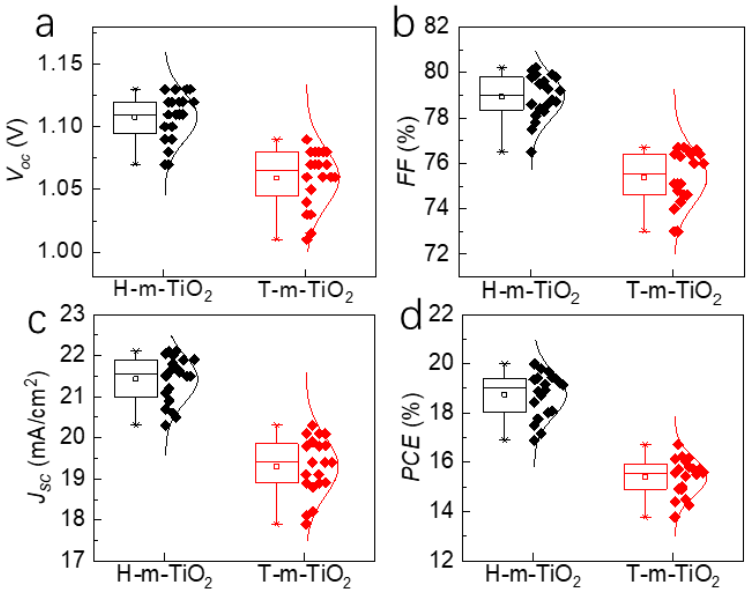

Figure S6. Histogram of device output parameters obtained from 20 H-m-TiO_2-based PSCs and 20 T-m-TiO_2-based PSCs: (a) V_{oc}, (b) FF, (c) J_{sc}, and (d) PCE, respectively.

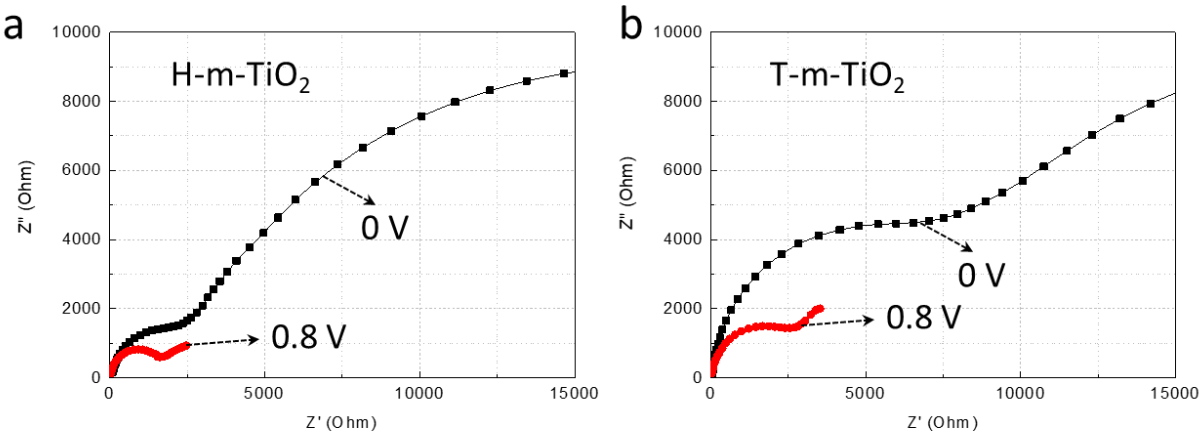

Figure S7. Nyquist plots with differet bias voltage for PSCs in (a) H-m-TiO_2 ETLs and (b) T-m-TiO_2 ETLs, respectively.

Table S1. The fitted EIS data of the PSCs based on H-m-TiO_2 and T-m-TiO_2, respectively.

PSCs	R_s (ohm)	R_{tr} (ohm)	R_{rec} (ohm)
Based on H-m-TiO_2	17.2	1980	~31000
Based on T-m-TiO_2	18.1	2300	~22500

Supplementary Note 1

Optical FDTD simulation. Optical simulation in perovskite solar ells were performed with 2-dimensional finite difference time domain (FDTD) algorithm. The total thickness of the simulated architecture was 1 μm. The wavelength-dependent optical constants for the TiO_2 and perovskite layers were carried out by ellipsometer measurement. The light was incidnet from the bottom of the architecture. The electric-field and in the simulated architecture were aquired from optical simulations.

Supplementary Note 2

Materials: Methylammonium (CH3NH2), Lead chloride (PbI2, 99.99%), hydroiodic acid (HI, 57 wt% in water), Spiro-OMeTAD, Anhydrous dimethyl sulfoxide (DMSO), and γ-butyrolactone were purchased from YOUXUAN Tech. The TiO_2 colloid were purchaused from Sigma Aldrich.

Supplementary Note 3

PSCs fabrication. A mixture solution of 462 mg PbI_2 /163 mg MAI dissolved in a Dimethyl sulfoxide (DMSO) and Dimethylformide (DMF) mixed solvent (v:v=3:7) under stirring at 70°C. The mixed solution (90μL) was spin-coated on the mesoporous TiO_2 film by a consecutive two-step spin-coating process at 1,000 and 5,000 rpm for 10 and 20 s. During the second spin-coating step, the substrate was treated with 400 μL chlorobenzene. Then the obtained films were dried at 100 °C for 10 min. All these as-prepared $MAPbI_3$ films were used to fabricate solar cells. We named as-prepared traditional $MAPbI_3$ films. A volume of 80 μL of the hole-transporting material was deposited onto the as-prepared $MAPbI_3$ films by spin coating at 3000 rpm for 30 s. The spin coating solution was prepared by dissolving 72.3 mg spiro-MeOTAD, 28.5 μL of a stock solution of 500 mg mL^{-1} lithium bis(trifluoromethylsulphonyl)imide in acetonitrile and 18.5μL 4-tert-butylpyridine in 1 mL chlorobenzene. Finally, a sliver counter electrode was deposited by thermal evaporation.

Supplementary Note 4

Characterization: The scanning electron microscope (SEM) images were performed using a Hitachi S-4800 equipped with an energy dispersive X-ray spectroscopy (EDS) system (EDAX Genesis 2000). The crystal structure of the samples was characterized by X-ray power diffraction (XRD) (Japan Rigaku D/max-ga X-ray diffractometer) using Cu Kα (λ=0.15406 nm). The photocurrent density dependence on the voltage (J–V) were measured under AM 1.5 G simulated sunlight illumination (100 mW/cm^2, Model 91160, Newport). The spectral response was taken by an EQE measurement system (QEX10, PV Measurement), which is equipped with a monochromator, a lock-in amplifier, a Xe lamp, and a current-voltage amplifier. The optical properties of the films were analyzed using an UV-Vis-near-infrared (NIR) spectrophotometer (UV-3101PC).
